# Establishing non-fasting reference values for plasma lipids levels based on age, sex, and puberty stage in a French-Canadian pediatric population

**DOI:** 10.1186/s12944-024-02040-0

**Published:** 2024-02-22

**Authors:** Sophie Bouhour, Rosalie Plantefève, Virginie Gillet, Armita Abolghasemi, Fatima Zahra Bouchouirab, Andrea A. Baccarelli, Larissa Takser, Artuela Çaku

**Affiliations:** 1 Department of Pediatrics, University of Sherbrooke, 3001 12E Avenue Nord, Sherbrooke, QC J1H 5N4 Canada; 2 Department of Biochemistry and Functional Genomic, University of Sherbrooke, Sherbrooke, QC Canada; 3 Department of Environmental Health Sciences, Columbia University Mailman School of Public Health, New York, NY USA; 4 Department of Psychiatry, University of Sherbrooke, Sherbrooke, Québec Canada

**Keywords:** Non-fasting state, Reference intervals, Pediatric, French-Canadian, Lipid profile, Dyslipidemia

## Abstract

**Background:**

Dyslipidemias, including familial hypercholesterolemia (FH), are a significant risk factor for cardiovascular diseases. FH is a genetic disorder resulting in elevated levels of low-density lipoprotein cholesterol (LDL-C) and an increased probability of early cardiovascular disorders. Heterozygous familial hypercholesterolemia (HeFH) is the most common form, affecting approximately 1 in 250 individuals worldwide, with a higher prevalence among the French-Canadian population. Childhood is a critical period for screening risk factors, but the recommendation for non-fasting screening remains controversial due to a lack of specific reference values for this state. This study aims to establish reference values for lipid levels in non-fasting children from Sherbrooke, Quebec, Canada, that will be specific for sex, age, and pubertal stages.

**Methods:**

Blood samples and corresponding anthropometric data were collected from 356 healthy children aged from 6 to 13. They were categorized either into two age groups: Cohort 6–8 and Cohort 9–13, or into pubertal stages. Reference values, specifically the 2.5th, 5th, 10th, 50th, 90th, 95th, and 97.5th percentiles were determined using the CLSI C28-A3 guidelines.

**Results:**

Lipid profiles did not significantly differ between sexes, except for higher levels of high-density lipoprotein (HDL-C) in boys within Cohort 6–8. HDL-C levels significantly increased, while LDL-C and non-HDL-C levels significantly decreased in both sexes with age. Non-fasting age- and pubertal stages-specific reference values were established.

**Conclusion:**

This study established reference intervals for lipid markers in non-fasting state within the pediatric French-Canadian population. These findings could be used in dyslipidemia screening in daily practice.

**Supplementary Information:**

The online version contains supplementary material available at 10.1186/s12944-024-02040-0.

## Introduction

Cardiovascular diseases (CVD) are a predominant global cause of mortality. According to the World Health Organization, ischemic heart disease and stroke account for 16% and 11% of the world’s total deaths, respectively [1]. In Canada, 2.6 million adults have been diagnosed with CVD [2], making heart diseases the second leading cause of death in 2022 [3]. Although CVD symptoms and complications appear in adulthood, the atherosclerotic process begins in childhood [4–6]. Dyslipidemia, a diagnosis used in clinical practice to characterise elevated levels of lipids in the blood, is recognized as a risk factor for CVD [7–9]. Screening of children for dyslipidemia allows for the identification of those who are at risk of developing CVD. Indeed, initiation of statin therapy in children with dyslipidemia aged between 8 and 18 years old, slows the progression of atherosclerotic process and reduces the risk of cardiovascular disease in adulthood. [10–14].

Familial hypercholesterolemia (FH) is the most common genic dyslipidemia causing premature CVD [15]^.^. FH is clinically characterized by high cholesterol levels, specifically high plasma levels of low-density lipoprotein cholesterol (LDL-C). Several mutations in genes encoding the low-density lipoprotein receptor (LDLR*)*, apolipoprotein B (ApoB*),* or Protein convertase subtilisin/kexin type 9 (PCSK9) account for the majority of FH cases [16, 17]. Heterozygous familial hypercholesterolemia (HeFH) is the most common form affecting 1 in 250 individuals worldwide [18]. The French-Canadian population has a higher prevalence of HeFH due to a founder effect, ranging from 1:80 to 1:270 [19].

The “Expert Panel on Integrated Guidelines for Cardiovascular Health and Risk Reduction in Children and Adolescents” released by the National Heart, Lung and Blood Institute (NHLBI) [20, 21] published guidelines for universal lipid screening in children and adolescents. Specifically, non-fasting lipid screening is recommended for all children aged 9 to 11 years without known risk factors. When established CVD risk factors are present, the screening should be carried out from the age of 1 year using a fasting lipid profile [20]. Since non-fasting measurements typically reflect normal physiology, they have greater clinical applicability [22]. LDL-C levels are used as diagnostic criteria for FH and as a biomarker of treatment efficacy of primary and secondary CVD prevention [23]. The LDL-C concentrations are often obtained by the Friedwald calculation [24–26]. However, the calculation is less accurate when plasma triglycerides (TG) are higher than 1,5 mmol/L, which is frequent in the postprandial state [27]. The use of non high-density lipoprotein (non-HDL-C) has been shown to be another reliable marker to estimate the risk of future cardiovascular events and is accurate even in the non-fasting state [28]. Altogether, it highlights the importance of obtaining reference values that are specifically tailored to the non-fasting state, particularly for non-HDL-C. The Canadian Cardiovascular Society (CCS) and the Canadian Pediatric Cardiology Association (CPCA) updated and approved NHBLI screening strategies [11]. Indeed, a non-fasting lipid profile is recommended and can be easily added to routine medical practices [11].

Although both Canada and the United States are North American countries, there are still specific environmental and genetic differences that could affect lipid levels. To ensure that physicians can correctly screen for dyslipidemia among children, it is crucial to have reference values adapted to the target population and for a non-fasting state. The Canadian Laboratory Initiative on Pediatric Reference Intervals (CALIPER) established age- and sex-specific reference intervals for non-fasting lipids, based on data from thousands of healthy children and adolescents [22, 29]. Those reference intervals are essential for the Canadian pediatric population. However, specific reference intervals are warranted for the French-Canadian pediatric population, considering its high prevalence of FH.

This study aimed to establish non-fasting reference values for total cholesterol (TC), HDL-C, non-HDL-C, LDL-C, TG, and ApoB for the French-Canadian pediatric population. Establishing reference values in a target population with a high prevalence of dyslipidemia will improve the ability of pediatricians to perform an early screening to detect children with a high risk of developing CVD.

## Subjects & methods

### Study population

The study population included participants from the GESTation and Environment (GESTE) cohort described previously [30–32]. Briefly, GESTE was a longitudinal study designed to investigate the impact of pollutant exposure during pregnancy on children’s neurodevelopment. GESTE comprised pregnant women from the Eastern Townships of Quebec, Canada, and children born from these pregnancies. The study population included 800 women recruited either during the first trimester of pregnancy (*n* = 400) or at delivery (*n* = 400), between 2007 and 2009 at the Research Center of the Centre Hospitalier Universitaire de Sherbrooke (CHUS). Inclusion criteria for women were: age over 18 years, absence of known thyroid disease, and no use of medications known to affect thyroid hormone levels. However, only the participants of the two last follow-ups were included in this study: the Cohort 6–8 (3rd follow-up) occurred when the children were aged 6 to 8 years old (2014–2016), and the Cohort 9–13 (4th follow up) was performed when children were at 9 to 13 years old (2018–2022). Children with medical conditions affecting lipid profile such as FH, diabetes, obesity, thyroid disease, malabsorption, positive family history of CVD, acute or chronic inflammatory conditions, or those taking medications known to alter lipid levels, were excluded. The inclusion and exclusion criteria of the children are summarized in Supplementary Table S1.

### Ethics

The study was approved by the Ethics Committee of both universities (2018–2542, University of Sherbrooke, Quebec, Canada, and Columbia University, New York, USA). Informed consent was obtained from both the children and their parents/legal guardians for all participants. The authors confirm that participants consent have been obtained for publication of this work.

### Lipid profile

To enhance the children's comfort, an anesthetic cream (such as EMLA®) was applied to the blood collection spot an hour before the procedure. Blood was collected during the day using EDTA BD Vacutainer®. The extracted plasma samples were stored at -80 °C, as previously reported [33]. Lipid parameters including TC, HDL-C, TG, and Apolipoprotein B (ApoB) were analyzed at the core lab of the CHUS. Specifically, TC, HDL-C, and TG were measured by enzymatic colorimetric methods, using a Modular/Roche-P800-analyser, following the manufacturer’s instructions, with the following commercial kits CHOL2, HDLC3 and TRIGL. The analytical parameters including total error, bias and precision met the National Cholesterol Education Program (NCEP) recommendations for TC, TG and HDL-C [34]. Apolipoprotein B was measured by immunoturbidimetric assays using a Cobas-6000-analyser with the commercial APOBT kit. ApoB data were available only for the Cohort 9–13.

LDL-C (mmol/L) was calculated using the Friedewald formula [27]: TC – HDL-C – (TG/2.2). To eliminate the impact of triglycerides on LDL-C calculation, the latter was not performed when TG was greater than 1.5 mmol/L [35]. The non-HDL-C (mmol/L) was calculated as TC – HDL-C.

### Anthropometric measures and Tanner stages

Anthropometric measurements including weight (measured using the Tanita Digital Scale TBF-300A model) and height, were obtained from all study participants. Children were instructed to wear light indoor clothing and remove their shoes. Body Mass Index (BMI) and BMI z-scores were then calculated using the '*anthroplus*' package in R, according to their heights and body weights (BMI = body weight [kg]/height^2^ [m]^2^). *Anthroplus* takes into account values from the “*Growth reference data for 5–19 years WHO chart”* [36, 37]. By obtaining the BMI z-scores, children were categorized as thin (< -2SD), normal (between -2SD and + 1SD), overweight (between + 1SD and + 2SD), or obese (> + 2SD) [38].

The participants of the Cohort 9–13 completed the self-administered Tanner scale questionnaire. Participants with a score of 1 on the Tanner scale were considered pre-pubertal, while those with a score of 2 to 5 were considered per-pubertal. For the Cohort 6–8, the pubertal stage questionnaire was not performed and the pubertal stage was assumed to be 1.

### Statistical analysis and establishment of the reference values

Demographic results were analyzed using appropriate statistical tests: Student's t-test or Wilcoxon test was applied based on the normality of the distribution for continuous variables, while Fisher's exact test was used for categorical data. To test the changes in lipid levels throughout growth, a paired t-test was used to compare lipid levels between the 3rd and 4th follow-ups, and an unpaired t-test was used to compare the two cohorts. The reference values were established based on the CLSI C28-A3 guidelines, as follows [39]. First, data underwent visual and statistical verification of distribution, using skewness statistics. As recommended by the CLSI C28-A3 guidelines, outlier data were identified with Tukey’s test, known for reducing the potential masking impact of outliers on one side of the distribution, and consequently removed [39, 40]. If log transformation was required, the data were transformed. Subsequently, the partitioning of data into groups, based on age, puberty stage, and sex was performed using Harris and Boyd's method [39, 41]. When partitioning between the two groups was not required, the data were merged into one group. When partition groups were greater than or equal to 120, the nonparametric rank method was used to determine the 2.5th, 5th, 10th, 50th, 90th, 95th, and 97.5th percentiles. The 95% confidence interval was determined only for the 2.5th and 97.5th percentiles, representing the lower and upper reference limits, respectively. When the partition group was less than 120, Horn and Pesce’s robust method [42] was used to determine the 2.5th and 97.5th percentiles. Statistical analyses were performed using Excel (*Microsoft,* version 16.81) and *RStudio* software (version 4.1.2, used for advanced functions and bootstraps).

## Results

### Design and population characteristics

**Fig. 1:**
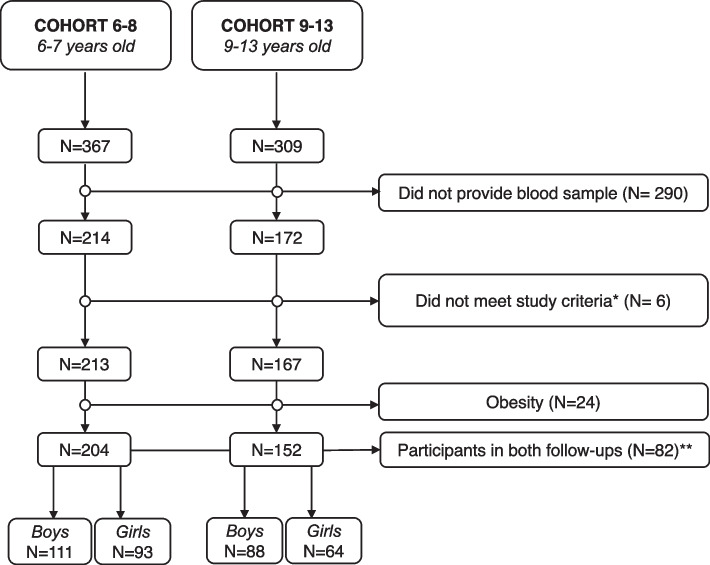
Flowchart for selection of study participants

The selection of study participants was performed as shown in Fig. 1. A total of 290 children refused to provide blood samples and 30 children did not meet the acceptability criteria: 6 were taking medications or had conditions affecting lipid levels and 24 had obesity. The final sample population was composed of 356 participants including 204 of Cohort 6–8 and 152 of Cohort 9–13. Our population included 199 boys and 157 girls. A total of 82 children participated in both follow-up visits (40 girls and 42 boys).

The demographic characteristics of study participants are summarized in Table 1. No differences were observed between the two cohorts for the gestational age at delivery (39.08 ± 1.43 vs 39.04 ± 1.48) and the ethnicity, with the majority being Caucasian. The mean age of Cohort 6–8 was 6.58 ± 0.51 years, while the mean age of Cohort 9–13 was 11.40 ± 1.04 years. The Cohort 6–8 had a significantly higher BMI compared to Cohort 9–13 (17.78 ± 2.38 vs 15.64 ± 1.20; *p* < 0.0001). However, both fell within the normal range. All children of Cohort 6–8 were in the pre-pubertal stage. The Cohort 9–13 had 56 children who described themselves in Tanner stage 1, and 95 in Tanner stages between 2 and 5. There were no significant differences between girls and boys for any of the demographic results.

**Table 1:** Characteristics of the study population

	**COHORT 6–8**				**COHORT 9–13**				*P-value *^*‡*^
	**Sex**		**Total**	*p-value* ^†^	**Sex**		**Total**	*p-value* ^†^	
	Boys	Girls			Boys (*N* = 88)	Girls (*N* = 64)			
	(*N* = 111)	(*N* = 93)							
**Age** Mean (SD)	6.58 (0.51)	6.59 (0.51)	6.58 (0.51)	ns*	11.31(1.03)	11.52 (1.05)	11.40(1.04)	ns*	< 0.0001*
**BMI** Mean (SD)	15.59 (1.10)	15.70 (1.32)	15.64 (1.20)	ns*	17.63 (2.37)	17.98 (2.33)	17.78 (2.36)	ns*	< 0.0001*
**Ethnicity**									ns***
African	3	0	3 (1%)	ns***	0	0	0 (0%)		ns***
Caucasian	106	91	197 (97%)	ns***	84	63	147 (97%)	ns***	ns***
Latin	1	2	3 (1%)	ns***	2	1	3 (2%)	ns***	ns***
South-Asian	1	0	1 (0.5%)	ns***	2	0	2 (1%)	ns***	ns***
**Gestational age at delivery** Mean (SD)	38.93 (1.58)	39.27 (1.21)	39.08 (1.43)	ns*	38.84 (1.54)	39.42 (1.32)	39.04 (1.48)	ns*	ns*
**Tanner Stage**									
1	111	93	204 (100%)	ns***	43	13	56 (37%)	< 0.001***	< 0.0001***
2	–	–	–		18	21	39 (26%)	*ns*	
3	–	–	–		20	19	39 (26%)	*ns*	
4	–	–	–		4	9	13 (9%)	*ns*	
5	–	–	–		2	2	4 (3%)	*ns*	
**Lipid Profile** Mean (SD)									
TC (mmol/L)	3.75 (0.57)	3.75 (0.56)	3.75 (0.56)	ns**	4.04 (0.63)	4.06 (0.56)	4.05 (0.60)	ns**	< 0.0001**
HDL-C (mmol/L)	0.68 (0.24)	0.60 (0.21)	0.65 (0.23)	< 0.01**	1.56 (0.28)	1.50 (0.31)	1.54 (0.30)	ns*	< 0.0001*
TG (mmol/L)	0.96 (0.45)	1.06 (0.54)	1.01 (0.49)	ns*	1.10 (0.63)	1.15 (0.60)	1.12 (0.62)	ns*	ns*
LDL-C(mmol/L)	2.63 (0.56)	2.72 (0.54)	2.67 (0.55)	ns**	1.93 (0.51)	2.10 (0.42)	2.00 (0.48)	ns**	< 0.0001**
Non-HDL-C (mmol/L)	3.06 (0.58)	3.15 (0.56)	3.10 (0.57)	ns**	2.47 (0.62)	2.56 (0.54)	2.51 (0.59)	ns*	< 0.0001**
ApoB (g/L)	–	–	–	–	0.70 (0.15)	0.71 (0.13)	0.71 (0.14)	ns*	–

*ApoB* Apolipoprotein B, *BMI* Body Mass Index, *HDL-C* High-density lipoprotein, *LDL-C* Low-density lipoprotein, *Non-HDL-C* Non-High-density lipoprotein, *SD* Standard Deviation, *TC* Total Cholesterol, *TG* Triglycerides

(*Wilcoxon rank sum test, **Two Sample t-test, *** Fisher's Exact Test

^†^ Comparisons between sex

^‡^ Comparison between Cohort 6–8 and Cohort 9–13

### Sex-based differences in lipid profiles

In our analysis, no significant differences were observed between boys and girls for TC, TG, LDL-C, ApoB, and non-HDL-C. The LDL-C analysis included a total of 300 samples with triglycerides (TG) levels less than 1.5 mmol/L. Boys displayed higher HDL-C levels as compared to girls in the Cohort 6–8 (0.68 ± 0.24 vs 0.60 ± 0.21, *p* < 0.01), while no differences were observed for the Cohort 9–13 (Table 1).

### Age-based differences in lipid profiles

Our analysis has shown significant differences in lipid levels according to age. When comparing the two cohorts, children of the Cohort 9–13 showed higher levels of TC (4.05 ± 0.60 mmol/L vs 3.75 ± 0.56 mmol/L; *p* < 0.0001) and HDL-C (1.54 ± 0.30 mmol/L vs 0.65 ± 0.23 mmol/L; *p* < 0.0001), but lower levels of LDL-C (2.00 ± 0.48 mmol/L vs 2.67 ± 0.55 mmol/L; *p* < 0.0001) and non-HDL-C (2.51 ± 0.59 mmol/L vs 3.10 ± 0.57 mmol/L; *p* < 0.0001).

**Fig. 2:**
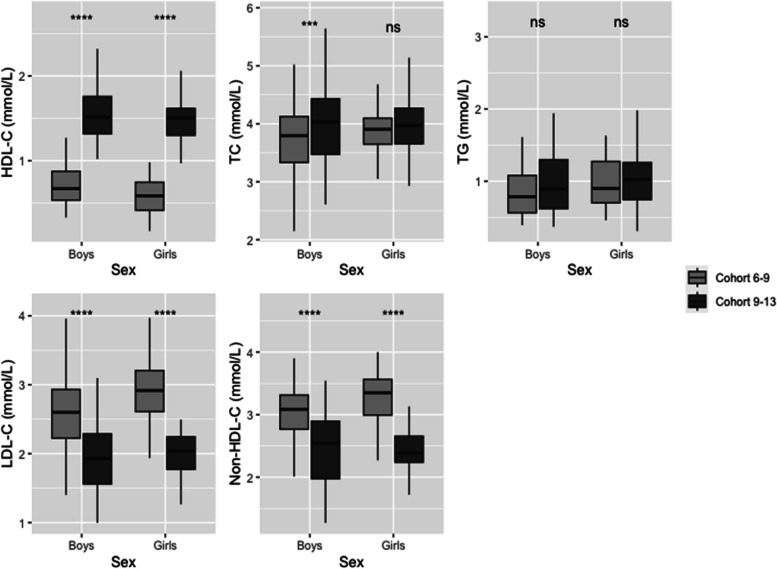
Impact of age on lipid levels for girls and boys. *HDL-C* High-density lipoprotein, *LDL-C* Low-density lipoprotein, *Non-HDL-C* Non-High-density lipoprotein, *SD* Standard Deviation, *TC* Total Cholesterol, *TG* Triglycerides

To confirm the changes in lipid parameters between the two cohorts, we analyzed data from a subset of 82 children who participated in both follow-ups (Tables 2 and 3). As shown in Fig. 2, a significant increase was observed for HDL-C between the two follow-ups. The HDL-C levels increased from 0.58 ± 0.21 mmol/L to 1.50 ± 0.27 mmol/L in girls, and from 0.71 ± 0.24 mmol/L to 1.58 ± 0.35 mmol/L in boys. In contrast, LDL-C and non-HDL-C levels significantly decreased in both girls and boys. The LDL-C decreased from 2.86 ± 0.46 mmol/L to 1.92 ± 0.74 mmol/L in girls, and from 2.56 ± 0.21 to 2.10 ± 0.20 mmol/L in boys. The non-HDL-C decreased from 3.29 ± 0.46 mmol/L to 2.48 ± 0.49 in girls, and from 3.03 ± 0.58 mmol/L to 2.45 ± 0.56 mmol/L in boys. TG levels remained consistent over time for both sexes. However, it is important to highlight that while TC levels remained constant in girls, there was a significant increase in TC levels among boys, rising from 3.74 ± 0.60 to 4.04 ± 0.65 mmol/L (*p* < 0.001). This trend persisted despite the exclusion of 82 children who participated in both follow-ups. Data are shown in Supplementary Tables S2 and S3.

**Table 2:** Age-related differences for girls who were present in Cohort 6–8 and in Cohort 9–13

	**Girls (*n* = 40)**		**Total**	*p-value*
	Cohort 6–8	Cohort 9–13		
**Age** (years) – Mean (SD)	6.49 (0.48)	11.49 (1.08)	8.99 (2.65)	< *0.0001*
**Lipid Profile**—Mean (SD)				
TC (mmol/L)	3.87 (0.40)	3.98 (0.53)	3.93 (0.47)	*ns*
HDL-C (mmol/L)	0.58 (0.21)	1.50 (0.27)	1.04 (0.52)	< *0.0001*
TG (mmol/L)	1.05 (0.49)	1.08 (0.47)	1.07 (0.48)	*ns*
LDL-C (mmol/L)	2.89 (0.46)	2.05 (0.43)	2.47 (0.61)	< *0.0001*
Non-HDL-C (mmol/L)	3.29 (0.46)	2.48 (0.49)	2.89 (0.63)	< *0.0001*

*HDL-C* High-density lipoprotein, *LDL-C* Low-density lipoprotein, *Non-HDL-C* Non-High-density lipoprotein, *SD* Standard Deviation, *TC* Total Cholesterol, *TG* Triglycerides

**Table 3:** Age-related differences for boys who were present in Cohort 6–8 and in Cohort 9–13

	**Boys (*n* = 42)**		**Total**	*p-value*
	Cohort 6–8	Cohort 9–13		
**Age** (years)—Mean (SD)	6.54 (0.49)	11.29 (1.00)	8.91 (2.52)	< *0.0001*
**Lipid Profile**—Mean (SD)				
TC (mmol/L)	3.74 (0.60)	4.04 (0.65)	3.89 (0.64)	< *0.001*
HDL-C (mmol/L)	0.71 (0.24)	1.58 (0.35)	1.15 (0.53)	< *0.0001*
Triglycerides (mmol/L)	0.83 (0.32)	1.05 (0.63)	0.94 (0.51)	*ns*
LDL-C (mmol/L)	2.57 (0.57)	1.95 (0.51)	2.26 (0.62)	< *0.0001*
Non-HDL-C (mmol/L)	3.03 (0.58)	2.45 (0.56)	2.74 (0.64)	< *0.0001*

*HDL-C* High-density lipoprotein, *LDL-C* Low-density lipoprotein, *Non-HDL-C* Non-High-density lipoprotein, *SD* Standard Deviation, *TC* Total Cholesterol, *TG* Triglycerides

Reference intervals for lipid parameters were established, including TC, HDL-C, LDL-C, and non-HDL-C (Table 4) specific for both, sex and age. Since TG did not require partitioning by sex and age, reference intervals for TG are provided separately (Table 5). Considering that ApoB levels were only measured for Cohort 9–13, a single reference interval was established from our analysis (Table 6).

**Table 4:** Age-specific reference intervals for TC, HDL-C, LDL-C and non-HDL-C in mmol/L

Analyte	Centiles	6 to 8 years	9 to 13 years
**TC (mmol/L)**	2.5 (CI)	2.59 (2.15—2.81)	2.89 (2.61—3.14)
	5	2.81	3.14
	10	3.01	3.22
	50	3.76	4.03
	90	4.47	4.87
	95	4.69	5.03
	97.5 (CI)	4.80 (4.69—5.03)	5.22 (4.98—5.64)
**HDL-C (mmol/L)**	2.5 (CI)	0.25 (0.19—0.30)	1.05 (0.98—1.10)
	5	0.3	1.1
	10	0.36	1.21
	50	0.65	1.51
	90	0.98	1.96
	95	1.05	2.1
	97.5 (CI)	1.16 (1.05—1.23)	2.22 (2.1—2.62)
**LDL-C (mmol/L)**	2.5 (CI)	1.5 (1.15—1.68)	1.02 (0.97—1.27)
	5	1.68	1.2
	10	1.94	1.36
	50	2.67	2.05
	90	3.37	2.65
	95	3.56	2.86
	97.5 (CI)	3.74 (3.55—3.97)	3.18 (2.73—3.26)
**Non-HDL-C (mmol/L)**	2.5 (CI)	1.83 (1.62—2.03)	1.41 (1.27—1.62)
	5	2.03	1.55
	10	2.42	1.77
	50	3.14	2.49
	90	3.83	3.32
	95	4.01	3.69
	97.5 (CI)	4.2 (4.01—4.54)	3.82 (3.57—3.89)

*CI* Confidence interval, *HDL-C* High-density lipoprotein, *LDL-C* Low-density lipoprotein, *Non-HDL-C* Non-High-density lipoprotein, *TC* Total Cholesterol

**Table 5:** Pediatric reference intervals for TG (mmol/L)

Analyte	Centiles	6 to 13 years
**TG (mmol/L)**	2.5 (CI)	0.39 (0.37—0.44)
	5	0.46
	10	0.52
	50	0.94
	90	1.69
	95	2.14
	97.5 (CI)	2.57 (2.25—2.92)

*CI* Confidence interval, *TG* Triglycerides

**Table 6:** Pediatric reference intervals for ApoB (g/L)

Analyte	Centiles	9 to 13 years
**ApoB (g/L)**	2.5 (CI)	0.43 (0.40—0.49)
	5	0.49
	10	0.55
	50	0.71
	90	0.92
	95	0.97
	97.5 (CI)	1.02 (0.97—1.08)

*ApoB* Apolipoprotein B, *CI* Confidence interval

### Pubertal-based reference values

Reference values were also established based on the pubertal stage and are shown in Table 7. Given that less than 120 children self-reported as Tanner stages 2 to 5, the reference values were obtained using the robust method recommended by the CLSI. The reference interval exhibited the same trend as the one partitioned based on age, except for TC, for which partition was not necessary.

**Table 7:** Pediatric reference intervals for lipid profiles, estimated by Tanner stages

Analyte	2.5th (CI)	97.5th (CI)
**TC (mmol/L)**	2.70 (2.58–2.82)	5.03 (4.92–5.22)
**TG (mmol/L)**	0.39 (0.37–0.44)	2.57 (2.25–2.92)
**ApoB (g/L)**	0.43 (0.40–0.49)	1.02 (0.97–1.08)
**HDL-C (mmol/L)**		
Tanner Stage 1	0.26 (0.20–0.31)	1.87 (1.82–2.10)
Tanner Stage 2–5	0.81 (0.69–0.92)	2.09 (1.96–2.21)
**LDL-C (mmol/L)**		
Tanner Stage 1	1.27 (1.00–1.41)	3.66 (3.55–3.96)
Tanner Stage 2–5	0.78 (0.66–0.91)	2.10 (1.96–2.23)
**Non-HDL-C (mmol/L)**		
Tanner Stage 1	1.70 (1.49–1.80)	4.05 (3.95–4.43)
Tanner Stage 2–5	1.23 (1.09–1.41)	3.60 (3.41–3.81)

*ApoB* Apolipoprotein B, *CI* Confidence interval, *HDL-C* High-density lipoprotein, *LDL-C* Low-density lipoprotein, *Non-HDL-C* Non-High-density lipoprotein, *TC* Total Cholesterol, *TG* Triglycerides

## Discussion

FH is a prevalent condition in the French-Canadian population [19]. Since childhood is a critical period for CVD risk screening, establishing non-fasting lipid reference values tailored the French-Canadian pediatric population, especially for the non-HDL-C, could enhance the implementation of routine dyslipidemia screening and CVD prevention. Indeed, the non-fasting testing not only increases the complience of patients, but also better reflects the effect of atherogenic lipoproteins [43]. However, non-fasting guidelines lack specific reference values for this population. The present study addressed this gap and provided reference intervals to facilitate accurate dyslipidemia screening. Our results confirm changes in lipid parameters according to age and puberty stage, particularly for HDL-C, LDL-C, and non-HDL-C.

Our findings indicate that sex does not have a significant impact on the lipid profile of children aged 6–13 years old, except for HDL-C, which was lower in girls as compared to boys. Nevertheless, previous studies reported sex-related differences. The CALIPER study determined reference values in a multiethnic Canadian pediatric cohort comprising 2188 children and adolescents (1072 male and 1116 female) aged 0–19 years [22, 29]. Their data required sex partitioning according to the CLSI guidelines for certain age groups for almost all non-fasting lipid parameters, including HDL-C, TC, non-HDL-C, LDL-C, and ApoB, highlighting the impact of sex on the lipid profile [22, 29]. Furthermore, the IDEFICS study identified significant sex disparities in fasting lipid parameters within a European population including 13,579 children aged 2 to 10 years [44]. They obtained lower HDL-C and higher TC, LDL-C, and TG levels in girls as compared to boys [44]. The Heartbeat! study [45] determined reference intervals for the fasting lipid profile of 633 white and black American children aged 8, 11, or 14 years old. They observed higher levels of all lipid parameters, including TC, LDL-C, and HDL-C in girls as compared to boys, for all age groups and pubertal stages [45]. Montazeri-Najafabady and colleagues [46] established reference intervals for the fasting lipid profile of 472 Iranian children aged from 9 to 18 years old. Their results showed higher levels of TC, LDL-C, and non-HDL-C in girls as compared to boys [46]. Altogether, this shows a lack of consistency among different studies regarding sex-related variations in lipid profiles, that can be explained by variations in sample size, ethnicity, age, pubertal stage, and fasting state.

In the present study, we investigated the effects of both, age and puberty on lipid profile. Our results showed an increase in HDL-C levels and a significant decrease in LDL-C and non-HDL-C levels from childhood (6–8 years) to adolescence (9–13 years old). A similar age partitioning was performed in the CALIPER study [22, 29]. TG remained stable between 1 and 19 years old, which is consistent with our study [29]. However, in contrast to our findings, they observed stable HDL-C levels for children younger than 13 years [29]. Moreover, their upper limits of LDL-C increased with age, especially after 10 years old [22]. The difference between population sample sizes might account for this discrepancy. In accordance with our results, Del Villar-Rubín and colleagues [47] showed a similar decrease in LDL-C levels of 356 healthy Spanish children from the age group 6–8 to 13–16 years.

Although pediatric studies report discrepancies in the variations of lipid parameters according to age, there is consensus on lipid changes during puberty. Consistent with our results, the German Health Survey for Children and Adolescents conducted on 13,676 European children and adolescents aged 1 to 17 years reported a decrease of LDL-C and non-HDL-C levels decreased with the progression of Tanner stages [48]. It is important to note that cholesterol is essential for cellular membrane structure and steroid synthesis. Cholesterol is the precursor of sex hormones such as estrogen, progesterone, and testosterone, which play a significant role in cellular and tissue growth [49]. Indeed, cholesterol involvement in body growth during the pubertal growth spurt and its utilization for hormone production might explain the decrease in plasma cholesterol levels during puberty [46, 48]. However, we were unable to validate this hypothesis since sex hormone levels were undetectable for the majority of children in our population. Hence, we could not conduct statistical analysis.

Recent guidelines such as the Canadian Cardiovascular Society and the Canadian Pediatric Cardiology Association recommended the use of non-fasting LDL-C and/or non-HDL-C as the initial step for pediatric dyslipidemia screening [11]. The non-fasting state is often associated with TG levels greater than 1,5 mmol/L, which underestimates the level of LDL-C [22]. Since fasting blood tests are difficult to perform in children and often require significant familial organization, physicians have to rely on non-HDL-C levels. The non-HDL-C reference values were suggested for dyslipidemia screening by the NCEP Expert Panel on Cholesterol Levels in Children [11, 20] and was subsequently adopted by the Canadian Cardiovascular Society [11]. Both NHBLI and the Canadian Cardiovascular Society recommend values under 3.4 mmol/L (130 mg/dL) and 3.75 mmol/L (145 mg/dL) for LDL-C and non-HDL-C, respectively, corresponding to the 95th percentile [11, 20]. The non-HDL-C values derived from the Bogalusa Heart Study that included 2843 American black and white children (50% female) aged 5 to 17 years old [20, 50]. In addition to sex- and ethnicity-specific percentiles, the latter study provided also distinct reference values for fasting children regardless the sex, age, ethnicity, or puberty stage [50]. In contrast, the CALIPER study established sex-specific thresholds for LDL-C and non-HDL-C corresponding to the 97.5th percentile of the population. In clinical practice, employing 2.5th and 97.5th percentiles as cut-off values for abnormal lipid levels implies that a total of 5% of healthy patients may exhibit values outside the normal range, leading to a misclassification of normal results as abnormal. Specifically, for LDL-C, they reported the following reference values: 3.14 mmol/L (CI: 3.02—3.35) for boys and 3.32 mmol/L (CI: 3.13—3.45) for girls under 10 years old, and 3.40 mmol/L (CI: 3.29—3.52) for both, boys and girls, aged 10 to 19 years old [22]. For non-HDL-C, they reported the following reference values: 3.68 mmol/L (CI: 3.62—4.08) for boys and 4.28 mmol/L (CI: 3.98—4.83) for girls under the age of 10 years, and 4.04 mmol/L (CI: 3.95—4.12) for both, boys and girls, aged 10 to 19 years old [22]. However, the proportion of French-Canadian participants has not been reported. Our reference threshold differs from the recommended values and CALIPER study. Our 97.5th percentile for children 6–8 years old is higher for LDL-C (3.74 mmol/L, CI: 3.55—3.97) and non-HDL-C (4.2 mmol/L, CI: 4.01 – 4.54). Although these reference intervals were considered high, several studies suggest that abnormal lipid levels tend to normalize with age, which is consistent with our findings [51, 52]. The difference might be due to ethnicity and fasting status.

Blood lipid concentrations are already known as a complex polygenic trait and a heritable risk factor for CVD. Data from 31 study cohorts conducted in 12 countries, encompassing a total of 24,760 men and 27,595 women aged 25–74 years showed that Asian Indian men and women had significantly higher likelihoods of low HDL-C compared to central and northern Europeans [53]. Moreover, a study on the Candidate Gene Association Resource (CARe) cohort, comprising 25,000 adults European Americans and 9000 adults African Americans, investigated the polymorphisms of 2000 genes and genetic loci from genome-wide association studies for association with lipid levels. The specific variants (LDLR and PCSK9 genes) related to plasma lipids differed between ethnic groups, specifically between the African Americans and the European Americans [54]. Another study comprising 5340 adults (2539 male and 2801 female) self-identified African Americans (*n* = 1355), Asian Americans (*n* = 666), Caucasians (*n* = 2063), and Hispanics Americans (*n* = 1256) from the Multi-Ethnic Study of Atherosclerosis found different associations between estimated genetic risk factors and lipid levels through ethnic populations [55]. Despite the genetic factor, the impact of ethnicity on lipid profile could also be due to population-variable factors such as diet, lifestyle, physical activity, weight, or smoking. Indeed, those are modifiable risk factors for CVD, that can also affect the lipid profile [55–58]. Altogether, that highlights the role of different ethnic profile on the lipid profile and emphasize that it need to be accounted for to have reference values tailored for each ethnicity.

### Strengths and limitations

The present work is the first study to establish reference values specifically for the French-Canadian pediatric population. The reference values, obtained in a non-fasting state, represent a first contribution towards the implementation of NHLBI and the Canadian Cardiovascular Society screening strategies within the routine examination. Moreover, this study also considered the impact of puberty on lipid level variations. Finally, the exclusion of children with known conditions that affect lipid metabolism ensures a population without known risk factors. However, there are a few limitations to consider. First, the sample size of our reference population was relatively small, which may limit our ability to conclude the necessity of sex-based partitioning. Though, the CLSI recommendation for subgroups with less than 120 participants were followed. Second, the age range of the study participants was limited, ranging from 6 to 13 years old. The latter did not fully capture the spectrum of puberty and hindered our ability to observe the variation of lipids during the later stages of puberty. Only four children identified themselves as being at Tanner stage 5, which prevented us from establishing reference values for each Tanner stage. Additionally, the Tanner scale questionnaire was not performed in the Cohort 6–8 and pubertal stage was assumed to be 1, a fact that might slithly affect the normal ranges according to pubertal stages. Third, this study did not include children younger than 6 years old, despite the Canadian Cardiovascular Society’s recommendation to initiate screening children at 2 years old if known risk factors for cardiovascular diseases exist within the family [11, 20]. Lastly, ApoB was measured at a single time point which made it impossible to follow its variation throughout puberty.

## Conclusions

To conclude, the present work is the first attempt to establish non-fasting reference intervals for TC, LDL-C, HDL-C, non-HDL-C, TG, and ApoB in a pediatric French-Canadian population. A significant increase in HDL-C levels combined to a significant decrease in LDL-C and non-HDL-C levels were observed across both sexes with age, confirming age- and puberty-related changes in lipid parameters. Given the high prevalence of FH in the French-Canadian population, tailored pediatric non-fasting lipid reference values may improve the implementation of routine dyslipidemia screening and contribute to more effective cardiovascular disease prevention. Local laboratories should validate these reference intervals before incorporating them into routine dyslipidemia screening practices for the French-Canadian population.

### Supplementary Information

**Additional file 1:** **Supplementary Table S1. **Inclusion and exclusion criteria for the participants. **Supplementary Table S2. **Age-related differences for girls excluding the 40 individuals present in both cohorts. **Supplementary Table S3. **Age-related differences for boys excluding the 42 individuals present in both cohorts.

## Data Availability

No datasets were generated or analysed during the current study.

## Abbreviations

ApoB: Apolipoprotein B
HDL-C: High-density lipoprotein
LDL-C: Low-density lipoprotein
Non-HDL-C: Non-High-density lipoprotein
TC: Total Cholesterol
TG: Triglycerides
