# Supplementary material for: Establishing non-fasting reference values for plasma lipids levels based on age, sex, and puberty stage in a French-Canadian pediatric population
Source: Lipids Health Dis. 2024 Feb 22;23:54. doi: 10.1186/s12944-024-02040-0 (PMC10882849; doi:10.1186/s12944-024-02040-0)
Supplement: Supplementary file 1 — Additional file 1: Supplementary Table S1. Inclusion and exclusion criteria for the participants. Supplementary Table S2. Age-related differences for girls excluding the 40 individuals present in both cohorts. Supplementary Table S3. Age-related differences for boys excluding the 42 individuals present in both cohorts. [file 12944_2024_2040_MOESM1_ESM.docx]

**SUPPLEMENTAL MATERIAL**

Establishing non-fasting reference values for plasma lipids levels based on age, sex, and puberty stage in a French-Canadian pediatric population

**Supplementary Table S1 –** Inclusion and exclusion criteria for the participants

**SUPPLEMENTAL RESULTS**

|  | Cohort 6-8 (n=53) | Cohort 9-13 (n=64) | Total | *p-value* |
| --- | --- | --- | --- | --- |
| **Age** (years) - Mean (SD) | 6.66 (0.54) | 11.52 (1.05) | 9.32 (2.58) | *<0.0001** |
| **Lipid Profile** - Mean (SD) | | | | |
| TC (mmol/L) | 3.66 (0.64) | 4.06 (0.56) | 3.88 (0.62) | *0.0004*** |
| HDL-C (mmol/L) | 0.62 (0.21) | 1.50 (0.31) | 1.10 (0.52) | *<0.0001*** |
| TG (mmol/L) | 1.07 (0.58) | 1.15 (0.60) | 1.11 (0.59) | *ns** |
| LDL-C(mmol/L) | 2.62 (0.57) | 2.10 (0.42) | 2.35 (0.56) | *<0.0001*** |
| Non-HDL-C (mmol/L) | 3.04 (0.60) | 2.56 (0.54) | 2.78 (0.61) | *<0.0001** |

**Supplementary Table S2 –** Age-related differences for girls excluding the 40 individuals present in both cohorts

(*Wilcoxon rank sum test, **Two Sample t-test)

HDL-C: High-density lipoprotein. LDL‑C: Low-density lipoprotein. Non-HDL-C: Non-High-density lipoprotein. SD: Standard Deviation. TC: Total Cholesterol. TG: Triglycerides

|  | Cohort 6-8 (n=69) | Cohort 9-13 (n=88) | Total | *p-value* |
| --- | --- | --- | --- | --- |
| **Age** (years) - Mean (SD) | 6.61 (0.52) | 11.31 (1.03) | 9.24 (2.48) | *<0.0001** |
| **Lipid Profile** - Mean (SD) | | | | |
| TC (mmol/L) | 3.75 (0.56) | 4.04 (0.63) | 3.91 (0.62) | 0.0040**** |
| HDL-C (mmol/L) | 0.67 (0.24) | 1.56 (0.28) | 1.17 (0.52) | *<0.0001** |
| TG (mmol/L) | 1.05 (0.49) | 1.10 (0.63) | 1.08 (0.57) | *ns** |
| LDL-C(mmol/L) | 2.61 (0.55) | 1.93 (0.51) | 2.24 (0.63) | *<0.0001*** |
| Non-HDL-C (mmol/L) | 3.09 (0.58) | 2.47 (0.62) | 2.74 (0.68) | *<0.0001*** |

**Supplementary Table S3 –** Age-related differences for boys excluding the 42 individuals present in both cohorts

(*Wilcoxon rank sum test, **Two Sample t-test)

HDL-C: High-density lipoprotein. LDL‑C: Low-density lipoprotein. Non-HDL-C: Non-High-density lipoprotein. SD: Standard Deviation. TC: Total Cholesterol. TG: Triglycerides
